# Screening the use of informed consent forms prior to procedures involving operative dentistry: ethical aspects

**DOI:** 10.15171/joddd.2017.013

**Published:** 2017-03-15

**Authors:** Livia Graziele Rodrigues, João Batista De Souza, Erica Miranda De Torres, Rhonan Ferreira Silva

**Affiliations:** ^1^Department of Dentistry, Federal University of Goias, Goiânia, Brazil

**Keywords:** Dental law, ethics, informed consent, operative dentistry

## Abstract

***Background. ***The present study aimed to screen the knowledge and attitudes of dentists toward the use of informed consent forms prior to procedures involving operative dentistry.

***Methods.*** A research tool containing questions (questionnaire) regarding the use of informed consent forms was developed. The questionnaire consisted of seven questions structured to screen the current practice in operative dentistry towards the use of informed consent forms.

***Results.*** The questionnaires were distributed among 731 dentists, of which 179 returned them with answers. Sixty-seven dentists reported not using informed consent forms. The main reasons for not using informed consent forms were: having a complete dental record signed by the patient (67.2%) and having a good relation with patients (43.6%). The dentists who reported using informed consent forms revealed that they obtained them from other dentists and made their own modifications (35.9%). Few dentists revealed contacting lawyers (1.7%) and experts in legal dentistry (0.9%) for the development of their informed consent forms.

***Conclusion.*** A high number of dentists working in the field of operative dentistry behave according to the ethical standards in the clinical practice, becoming unprotected against ethical and legal actions.

## Introduction

The dental record comprehends a valuable clinical tool that consists of a compilation of the documents and information necessary to the administrative, clinical and legal procedures in dentistry.^1^These documents may include the clinical file, medical prescriptions and certificates, complimentary imaging exams (e.g. radiographs), and informed consent forms.^2^ The dental record of each patient may be handled manually or digitally, by a single dentist or several dentists, becoming potentially misfiled during this process.^3^ According to the Brazilian Code of Dental Ethics, the dentist must be aware of properly implementing clinical data in the patient’s dental record.^4^ The same code also considers as ethical fault the lack of giving to the patient treatment alternatives (when applicable) and proper explanations on the treatment objective, risks and costs.^4^

In this context, informed consent plays an essential part in the routine of dentistry. Ideally, this document contains information related to the treatment steps, as well its benefits, risks and limitations.^5^ The informed consent should be structured, avoiding technical writing, allowing the patient to understand the treatment.^6,7^After carefully reading and understanding this document, the patient should voluntarily sign it, agreeing with the exposed information.^8^ Clearly, the informed consent represents a symbol of autonomy given to the patient in relation to the dental treatment,^9,10^and contributes to a better relation between the patient and dentist.^11,12^

In specific fields of Dentistry, the expectations for clinical outcomes are more pronounced, such as aesthetic treatments in operative dentistry. The dentists involved in these fields should be necessarily more cautious against potential conflicts between the obtained and the expected clinical outcomes.^13^ Over the last decade, the number of lawsuits filed in these conflicts considerably increased in Brazil.^14^ The informed consent arose as a mechanism to protect dentists from legal demands^5^ and to control the patients’ expectations for clinical outcomes. The present study aimed to screen the use of informed consent prior to procedures involving operative dentistry, highlighting the importance given by the dentists to this useful tool in the dental armamentarium against legal conflicts.

## Methods

The present study was approved the Committee of Ethics in Research of the Federal University of Goias, Brazil, under the protocol number 741.338/2014.

A research tool containing questions (questionnaire) regarding the use of informed consent was developed. The questionnaire had seven self-applicable questions structured to screen the current practice in operative dentistry in relation to the use of informed consent. The questionnaire was handed in to dentists working within the field of operative dentistry, properly registered in the Regional Council of Dentistry of Goias (CRO-GO) or in the Brazilian Society of Aesthetic Dentistry (SBOE). Seven hundred thirty-one dentists were contacted. Their personal data were not consulted, assuring confidentiality.

**Figure 1.**
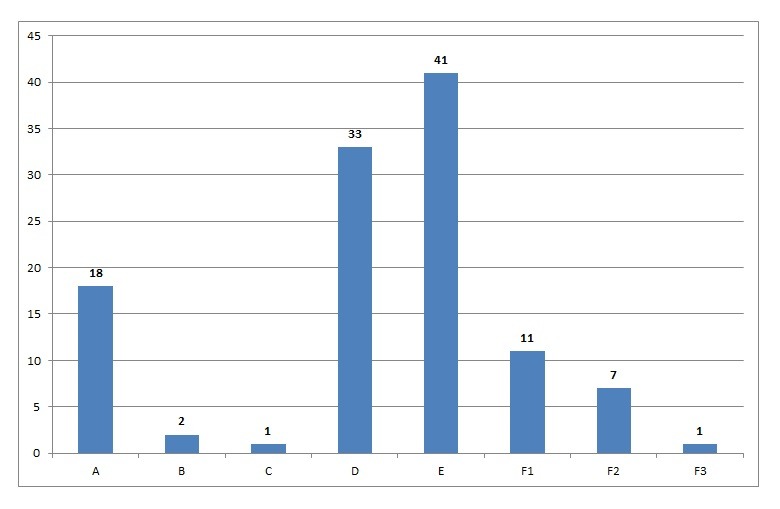
Number of dentists distributed according to the answer for the question “*How did you obtain your informed consent?*”A: I found in the internet; B: I contacted a lawyer; C:I contacted a specialist in Legal Dentistry; D: I designed it myself; E: I obtained from a colleague and made my own adaptations: F: other (F1: I obtained this document in a graduation course/training; F2: I work in a clinic that already had this document; F3: I adapted a** version from a scientific publication).**

The questions and possible answers were: 1) Dentist’s sex (possible answers: male/female); 2) Do you provide verbal explanations on the treatment plan? (possible answers: yes/no); 3) Do you use informed consent forms in operative dentistry? (possible answers: yes, every time/yes, sometimes/no, I do not see the need for bureaucratizing the clinical routine/no, the patient is not able to understand what is written in the informed consent/no, the informed consent has no legal value/no, I use a treatment contract instead of the informed consent/no, I have a good relationship with my patients/no, I am insured for civil liability/no, I have complete dental records signed by the patient/no, I am supported by lawyers); 4) How did you obtain your informed consent form? (possible answers: I found it on the internet/I contacted a lawyer/I contacted a specialist in legal dentistry/I designed it myself/I obtained it from a colleague and made my own adaptations/other); 5) When do you apply the informed consent form to the patient? (possible answers: in the first consultation, before starting the treatment/in the first consultation but after starting the treatment/other); 6) Who applies the informed consent form in your office? (possible answers: I apply it/my secretary or staff members apply); 7) Do you believe that the patient understands all the information contained in your informed consent? (possible answers: yes/no). In case of answering “other” in questions 4 and 5, the dentist had to describe any other answer not included among the possible options (Figure 1).

The data obtained underwent descriptive statistics using SPSS^®^ Statistics 21 (IBM^®^ Corp., Armonk, New York, USA) software package. The association between the dentists’ sex and the other variables was performed using chi-squared test following a significance level of 5% (P < 0.05).

## Results

One hundred seventy-nine (24.5%) out of 731 dentists answered the questionnaire.

**Question 1:** Seventy-one dentists were male (39.7%) and 108 (60.3%) were female.

**Question 2:** One hundred seventy-one (95.5%) dentists revealed providing verbal explanations on the treatment plan for their patients.

**Question 3:** Twenty-six (14.5%) dentists confirmed using informed consent forms every time in the dental practice. Eighty-six (48%) dentists revealed using informed consent forms sometimes, only in special cases. Sixty-seven (37.5%) dentists did not use informed consent forms in the clinical routine. Fifty-five (82.08%) of these dentists justified the lack of informed consent forms in their clinical routine. Most of them stated having complete dental records signed by the patients (n= 37; 67.2%) or having a good relation with their patients (n=24; 43.6%; Table 1).

**Table 1:** Reasons for not using the informed consent in the clinical practice (n=55)

**Reason**	**# (%)**
I do not see the need for bureaucratizing the clinical routine	6 (10.5%)
The patient is not able to understand what is written in the informed consent	5 (9.0%)
The informed consent has no legal value	0 (0.0%)
I use a treatment contract instead of the informed consent	7 (12.7%)
I have a good relationship with my patients	24 (43.6%)
I am insured for civil liability	3 (5.4%)
I have a complete dental records signed by the patient	37 (67.2%)
I am supported by lawyers	4 (7.2%)

#: number of dentists; %: percentage of dentists

**Question 4:** Considering the dentists that answered how they obtained their informed consent forms (n=114), 41 (35.96%) obtained them from their colleagues and made their own modifications, while 33 (28.94%) designed their own informed consents.

**Question 5:** One hundred (86.8%) dentists used informed consent forms in the first consultation, before starting the treatment; 6 (5.3%) dentists used them in the first consultation after starting the treatment; and 9 (7.9%) dentists answered “other”, using them prior to the agreement and signing of the treatment plan.

**Question 6:** Ninety-seven dentists (85.8%) applied informed consent forms themselves. The remaining dentists (n=16; 14.2%) delegated this task to the secretary or staff members.

**Question 7:** Sixty (51.3%) dentists believed that the patients understand the information contained in the informed consent, while 57 (48.7%) dentists believed the opposite.

Statistically significant associations were found between the dentists’ sex and other variables (P < 0.05).

## Discussion

The Brazilian Code of Dental Ethics establishes as mandatory the development and storage of complete dental records.^4^ Despite this, 67 (37.5%) dentists participating in the present study revealed not using informed consent forms in the routine of their clinical practice. From an ethical point of view, an informed consent form represents an essential part of dental records. Consequently, a lack of this document is a violation of ethical principles.

An informed consent form is a written document that aims to clarify the treatment plan for the patient. This document must be signed by the patient in case of agreement. However, the agreement is only reached properly when the patient is fully aware of the treatment steps, benefits, risks and limitations. Based on that, the informed consent form should be followed by verbal explanations, making the information more understandable for the patient. In the present research, 8 (4.5%) dentists did not provide verbal explanations. On the other hand, 60 (33.5%) dentists provided verbal explanations but did not use the informed consent form. As previously mentioned, the best practice in dentistry consists of the combination of using an informed consent form followed by verbal explanations on it. Providing exclusive verbal explanations does not exempt the dentist from the obligation of providing a written informed consent form.^11^ Yet the lack of using an informed consent form in the clinical practice is translated as a major vulnerability for ethical and legal penalties to which the dentist is exposed.

The most referred reasons to justify the lack of using informed consent forms were “having complete dental records signed by the patient” and “having a good relationship with the patients”, which were answered by 37 (55.2%) and 24 (35.8%) dentists, respectively. However, both reasons do not replace ethically the role a signed informed consent form. Moreover, 3 (4.4%) dentists reported not using informed consent forms because they were insured for civil liability. It reveals that the dentists are searching for a safer clinical practice. However, the insurance for civil liability is not useful to defend the dentist against legal actions, but yet exclusively to cover financial penalties in case of conviction.

Furthermore, the dentists were asked about how they obtained their informed consent forms. Ideally, this document should be designed by professionals experienced in dental laws and ethics, such as lawyers and specialists in legal dentistry. However, most of the dentists (n=41; 35.9%) obtained their informed consent forms from colleagues and made their own modifications, or designed their own documents (n=33; 29%). Lawyers and specialists in legal dentistry were contacted in less than 3% of the cases (n=2; n=1, respectively). The very low number (n=1) of dentists that contacted a specialist in legal dentistry highlights the fact that the dentists working in the field of operative dentistry are not completely aware of the existence of a legal specialty in dentistry. Specifically, the legal/forensic dentists are trained to instruct and guide the clinical routine towards the best ethical practice. It includes the development of informed consent forms, written adequately for the patients.

The Brazilian Code of Dental Ethics mandates that starting any treatment procedure without the patients’ consent, or without explaining its benefits, risks and limitations, constitutes a breach of the ethical principles.^4^ Accordingly, 100 (86.8%) dentists revealed using informed consent forms in the first consultation before starting the treatment. This practice is considered correct because it allows the patient to read and understand the informed consent form. Moreover, the dentists are encouraged to provide a copy of this document to the patients. So, this copy can be revisited by the patient and read out of the dental office.

Another aspect of relevance observed in the present study was the percentage of dentists (n=16; 9.1%) that delegated the application of the informed consent form to the secretary or staff members. This attitude does not coincide with the Brazilian Code of Dental Ethics, which considers as a breach of the ethical principles the delegation of procedures to persons without a formal and technical education in the field (undergraduation in Dentistry).^4^ This ethical aspect aims to support the clinical practice, especially in cases involving patients unsatisfied with the treatment. In these cases, the patients may claim that they did not receive proper explanations on the informed consent form or even sue the dentists for delegating this task to other persons.

The present study revealed some aspects that may interfere with the interpretation of the informed consent form, such as the application of the informed consent followed by more detailed verbal explanations; the time provided for the patient to read and understand the document; and the application of the informed consent form by a dentist. These aspects are corroborated by the last question of the present study, which showed that nearly 50% (n=57) of the dentists believed that the patients did not interpret or understand the content exposed in the informed consent form correctly. In this context, dentists must be sensitive to realize when the patients are not assimilating the information provided for their consent. In these cases, the dentist must change their approach, adopting a new way to explain the treatment steps, benefits, risks and limitations.^6,7^ It certainly minimizes the potential conflicts that may arise in the close relation between dentists and patients.

## Conclusion

Only 14.5% of the surveyed dentists used informed consent forms routinely in the clinical practice. Less than 3% of the dentists contacted a lawyer or a specialist in legal dentistry for developing their informed consent forms. These findings indicate that most of the dentists ignore the importance of legal documents, becoming exposed to ethical and civil conflicts in courts over time.

## Acknowledgments

The authors express their gratitude to the Brazilian Dental Association (ABO – Goias section), Prof. Rafael Decurcio and Prof. Fernanda Maria de Castro for strongly supporting the present study.

## Authors’ contributions

LG conducted the survey of data; EM performed statistical evaluations; LG and RF interpreted the data and drafted the manuscript; and JB made critical analysis of the manuscript. All the authors have read and approved the manuscript end.

## Funding

There was no funding for this research.

## Competing interests

The authors declare no competing interests with regards to the authorship and/or publication of this article.

## Ethics approval

The present study was approved the Committee of Ethics in Research of the Federal University of Goias, Brazil, under the protocol number 741.338/2014.
